# Aspirin is associated with a reduction in mortality rate for patients with sepsis-induced coagulopathy: a retrospective cohort study

**DOI:** 10.3389/fphar.2025.1537994

**Published:** 2025-07-28

**Authors:** Dan Xu, Jingyuan Li, Zhiyuan Wang, Junda Li, Qingyu Zhao, Qiannan Zhao, Fei Xie, Tingting Li, Jiying Chen, Xiya Wang, Xin Zhou, Yuan Guo, Shuxing Wei

**Affiliations:** ^1^ Department of General Practice, Qilu Hospital, Cheeloo College of Medicine, Shandong University, Jinan, Shandong, China; ^2^ The Key Laboratory of Cardiovascular Remodelling and Function Research, Chinese Ministry of Education and Chinese Ministry of Public Health, Department of Cardiology, Qilu Hospital of Shandong University, Jinan, China; ^3^ Shandong Medicine and Health Key Laboratory of Emergency Medicine, Department of Critical Care Medicine, Shandong Institute of Anesthesia and Respiratory Critical Medicine, The First Affiliated Hospital of Shandong First Medical University and Shandong Provincial Qianfoshan Hospital, Jinan, China; ^4^ Department of Critical Care Medicine, Shandong Provincial Hospital Affiliated to Shandong First Medical University, Jinan, China

**Keywords:** sepsis-induced coagulopathy, aspirin, medical information marketplace for intensive care database, propensity score matching, mortality

## Abstract

**Background:**

This study aimed to examine whether aspirin reduces mortality in patients with sepsis-induced coagulopathy (SIC).

**Methods:**

In this retrospective cohort study, 1,194 patients with SIC were identified from the Medical Information Mart for Intensive Care (MIMIC)-IV database. The primary outcome was 28-day all-cause mortality. Secondary outcomes included 90-day and 1-year all-cause mortality, as well as length of stay in the intensive care unit (ICU). Missing data were handled using multiple imputation, and baseline differences between groups were adjusted through propensity score matching (PSM). The association between aspirin therapy and mortality in SIC patients was evaluated using both univariate and multivariate Cox proportional hazards models. Additionally, subgroup analyses were performed to investigate the effect of aspirin across different populations and to assess the impact of aspirin dosage on clinical outcomes. External validation was subsequently conducted to confirm the robustness of the findings.

**Results:**

After PSM, 280 aspirin-treated patients were matched with 280 non-aspirin patients. Aspirin use was associated with significantly lower 28-day mortality (11.8% vs. 29.3%, *p* < 0.001), 90-day mortality (16.8% vs. 33.6%, *p* < 0.001), and 1-year mortality (22.1% vs. 42.1%, *p* < 0.001), as well as a shorter median ICU stay (2.19 vs. 3.14 days, *p* < 0.001) among patients with SIC. Multivariate Cox regression further confirmed the protective effect of aspirin on 28-day (hazard ratio [HR]: 0.45, 95% confidence interval [CI]: 0.29–0.7), 90-day (HR: 0.55, 95% CI: 0.37–0.81), and 1-year mortality (HR: 0.59, 95% CI: 0.42–0.83). Additionally, when comparing the efficacy of low-*versus* high-dose aspirin therapy, the low-dose group demonstrated significantly lower 28-day, 90-day, and 1-year mortality rates. External validation further supported these findings, showing reduced 28-day mortality (15.3% vs. 35.9%, *p* = 0.01) and improved overall survival (*p* = 0.0037) in the aspirin-treated group.

**Conclusion:**

Aspirin use was associated with reduced 28-day, 90-day, and 1-year mortality, as well as a shorter ICU stay in patients with SIC. These findings were confirmed through external validation.

## 1 Introduction

Sepsis is an abnormal host response to infection that leads to multiple organ dysfunction (Singer et al., 2016). Epidemiological studies indicate that sepsis affects millions of people worldwide each year and accounts for 20% of all global deaths (Rudd et al., 2020). Sepsis-induced coagulopathy (SIC) is a kind of vascular endothelial cell injury and coagulation disorder caused by sepsis (Levi and van der Poll, 2017). Infection is the main inducer in the progression of SIC. In the initial stage of sepsis, coagulation works as a natural defense, limiting and preventing the spread of pathogens into the systemic circulation (Iba et al., 2020). However, in the terminal and severe stage of sepsis, the excessive inflammatory cytokines in the circulation can lead to activated coagulation, impaired fibrinolysis, and inhibition of anticoagulants. Approximately 44% of patients with sepsis experience SIC, which remains a primary cause contributing to multiple organ failure and mortality in patients with sepsis (Schmoch et al., 2023). In 2017, an international committee introduced the term SIC (Iba et al., 2017; Yamakawa et al., 2019). The diagnostic criteria for SIC include platelet count, international normalized ratio, and total Sequential Organ Failure Assessment (SOFA) (Iba et al., 2017). In clinical practice, approximately 50%–70% of patients with sepsis will develop hemorrhagic disorders, and among them, 35% will develop disseminated intravascular coagulation (DIC) (Tsantes et al., 2023). DIC is characterized by the dysfunction of the coagulation cascade leading to intravascular fibrin formation, microangiopathic thrombosis, and subsequent depletion of coagulation factors and platelets, resulting in excessive thrombosis and bleeding complications (Yamakawa et al., 2013). Although sepsis is a common cause of DIC, other pathophysiological conditions, including trauma, cardiogenic shock, or acute ischemic injury, can also develop DIC. The diagnostic criteria for overt DIC and SIC, proposed by the International Society on Thrombosis and Haemostasis (ISTH) indicated that the sensitivity of the SIC score is twice that of the overt DIC score, and SIC always precedes overt DIC (Iba et al., 2017; Yamakawa et al., 2019). The coagulopathy in sepsis patients is initially compensatory disorder (as non-overt DIC), if not treated properly, it may develop into a fully decompensated coagulopathic state (overt DIC). Therefore, early identification of patients with SIC is an ideal period of time for treatment. At present, the treatment of SIC mainly follows anticoagulation, anti-inflammation, and anti-infection, while improving outcomes of SIC remains a critical healthcare challenge.

Aspirin, also known as acetylsalicylic acid, is a common pain reliever that helps reduce fever, inflammation, and blood clotting, mainly used to prevent heart disease (Smith et al., 2011). Aspirin inhibits cyclooxygenase (COX)-1 and COX-2 enzymes, reducing prostaglandin and thromboxane production, thereby preventing platelet aggregation and exerting antithrombotic effects (Vane and Botting, 1997). SIC is characterized by thrombin-driven hypercoagulability and platelet activation. Microvascular thrombosis (not bleeding) is the primary driver of organ failure in SIC. Aspirin inhibits COX-1, thereby targeting platelet-derived thromboxane A2 and neutrophil extracellular traps (NETs), both of which are key mediators in SIC-associated organ failure. The relationship between aspirin and sepsis outcomes has been explored in various studies, indicating that aspirin may play a beneficial role in managing sepsis-related complications. A study has suggested that aspirin may reduce the risk of death in patients with sepsis, especially in patients complicated with acute kidney injury (Chen J. et al., 2023). Furthermore, the anti-inflammatory properties of aspirin may be related to the alleviation of multi-organ dysfunction caused by sepsis (Otto et al., 2013). Inflammation and coagulation are influenced by activated platelets; therefore, inhibiting activated platelets and reducing the interaction of platelet-inflammatory cell-endothelial cells may block the cascade process of inflammation and coagulation (Wang et al., 2018). A study by Carestia et al. expressed that acetylsalicylic acid inhibits intravascular coagulation during *Staphylococcus* aureus-induced sepsis in mice, supporting the potential therapeutic role of aspirin for SIC (Carestia et al., 2020). Considering that patients with SIC are accompanied by severe bleeding and thrombotic complications, aspirin use may serve as a potential therapeutic agent (Meziani et al., 2024).

At present, the evidence-based basis of aspirin in the treatment of SIC has not been completely clear at home and abroad, furthermore, the timing and dose of aspirin for SIC treatment are still unclear. Therefore, this study was designed to explore the relationship between aspirin and the mortality of patients with SIC. However, future clinical trials should focus on the potential benefits of aspirin in septic patients in order to provide more explicit guidance in its clinical application (Inata, 2020).

## 2 Materials and methods

### 2.1 Data source

This study utilized the MIMIC-IV database (version 2.2) (Johnson et al., 2023), a public resource comprising data from 2008 to 2019 on patients admitted to the intensive care unit (ICU) at the Beth Israel Deaconess Medical Center in Boston, Massachusetts. This dataset included patient demographic information, medical history, vital signs, laboratory results, drug prescriptions, nursing records, and medical imaging reports. A doctor completed the Collaborative Institutional Training Initiative’s training and examination to access the MIMIC-IV database (certification number: 64114319). All data were fully anonymous, and informed consent was not required.

### 2.2 Study population

Enrolled patients were diagnosed with SIC within 24 h of intensive care unit admission. Sepsis was defined using the Sepsis-3 criteria (Singer et al., 2016), while SIC was identified based on the Sepsis-3 guidelines, while SIC was assessed using Toshiaki Iba’s scoring system, which includes prothrombin time (PT), international normalized ratio (INR), platelet (PLT) count, and SOFA score. Patients using anticoagulant agents and those with tumors, poisoning, cirrhosis, or antiphospholipid syndrome were excluded.

### 2.3 Exposure and outcomes

Aspirin usage during the ICU stay was the exposure variable. The standard for aspirin dosage regarded taking 81 mg or less per day as a low dose, while higher amounts were considered a high dose. This criterion was chosen based on previous studies (Eisen, 2012; Dong et al., 2024). The primary endpoint analyzed was 28-day all-cause mortality, with the secondary endpoints including 90-day and 1-year all-cause mortality, as well as the duration of ICU stay.

### 2.4 Data extraction

We utilized PostgreSQL (version 14.2) and Structured Query Language to access baseline characteristics, encompassing patient demographics (sex, age, and race), vital signs (HR-heart rate, MAP-mean arterial pressure, SpO_2_-oxygen saturation of hemoglobin, and RR-respiratory rate), laboratory test results (PLT-platelets, WBC-white blood cell count, BUN-blood urea nitrogen, Cr-creatinine, APTT-activated partial thromboplastin time, PT-prothrombin time, INR-international normalized ratio, and lac-lactate), comorbidities (DM-diabetes, CAD-coronary artery disease, CKD-chronic kidney disease, AKI-acute kidney injury and hypertension), intervention (vasopressor, CRRT-continuous renal replacement therapy, and MV-mechanical ventilation), the length of ICU stay, SIC score, and SOFA score.

### 2.5 Statistical analysis

In our study, the missing rate of variables was <10% (Supplementary Figure S1). We estimated missing values by using multiple imputations. A propensity score matching (PSM) analysis was performed to adjust for variables such as age, sex, race, CAD, SIC, CKD, SOFA score, MV, vasoactive agents, lactate, and CRRT. The matching process used a 1:1 ratio and the nearest neighbor method, with a caliper width of 0.1 and without replacement. Continuous data were compared using either a Mann–Whitney U test or an independent samples t-test, depending on what was appropriate, and the results were shown as mean ± standard deviation or median (interquartile range). Categorical variables were described as percentages and compared using the chi-square test. To visualize the cumulative mortality over a 28-day, 90-day, and 1-year period, Kaplan–Meier curves were used. Group differences were evaluated using the log-rank test. Univariate and multivariate Cox regression models were used to determine the effects of aspirin use on SIC outcomes. A multivariate Cox regression analysis was conducted for subgroup evaluation. We used the R software (v.4.4.1) to conduct all statistical analyses. Statistical significance was determined at *p* < 0.05.

### 2.6 External validation

An external validation was conducted using data from Qilu Hospital of Shandong University, comprising patients in the ICU with SIC from January 2021 to May 2024, to validate the primary endpoints. This study received approval from the hospital’s ethics committee (Approval number: KYLL-202404-061-2). To minimize confounding factors, a multivariate Cox regression model was employed to examine the relationship between aspirin administration and the 28-day mortality.

### 2.7 Post-hoc power calculation

Post-hoc power analyses were conducted on the after-PSM cohort. For the multivariable Cox regression-based calculation, Schoenfeld’s formula ( $D=Z_{1-\alpha }+Z_{1-\beta }^{2}P1-\text{Plog}{∆}^{2-1}$ ) was applied using the total number of death events (Hsieh and Lavori, 2000), the proportion of patients in the aspirin group, the observed hazard ratio, and a two-sided α of 0.05. For the log-rank test–based calculation, the R pwr package was employed, with inputs consisting of the 28-day mortality rates in the aspirin and control groups, their respective sample sizes, and a two-sided α of 0.05. All calculated power values substantially exceeded the conventional threshold of 0.80, indicating that the study was adequately powered to detect the observed differences in survival across time points (Supplementary Table 1).

## 3 Results

### 3.1 Patient characteristics

**FIGURE 1:**
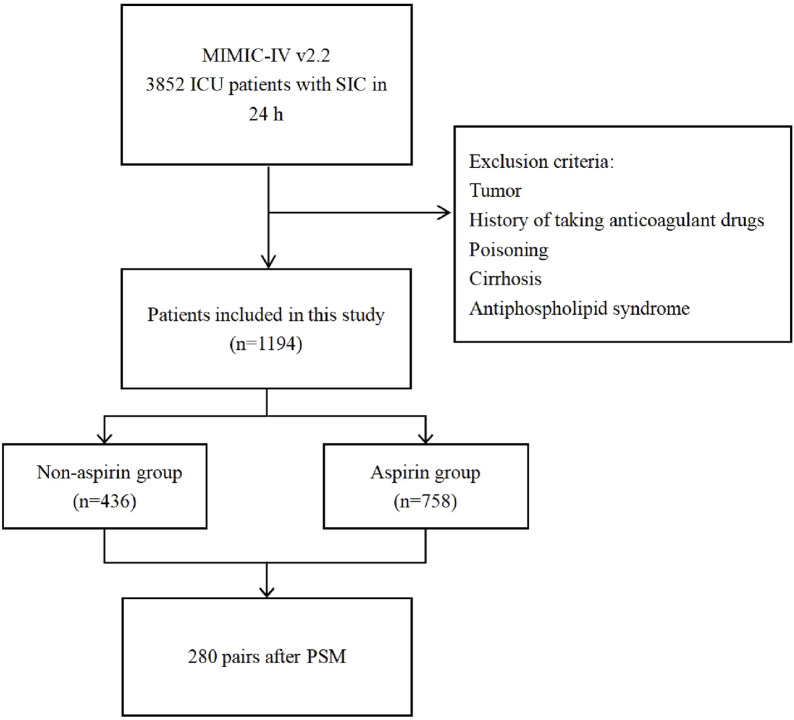
Flow chart of this study.

From the MIMIC-IV database, 3,852 patients who were diagnosed with SIC and admitted to the ICU within 24 h were enrolled in this study. Following the exclusion criteria, 1,194 patients were eligible for this study cohort. Among them, 758 individuals received aspirin, while 436 individuals did not. Based on aspirin use, patients included were categorized into the aspirin and non-aspirin groups, respectively (Figure 1).

Table 1 presents the patients’ baseline characteristics. Significant differences were observed between the aspirin and non-aspirin groups in terms of age, sex, race, RR, MAP, heart rate, SpO_2_, Cr, BUN, PLT, lactate, APTT, INR, vasopressors, MV, CRRT, AKI, hypertension, ICU stay, SIC, and SOFA score. According to the comparison between the two groups, patients who took aspirin were generally older (69.40 ± 12.18 vs. 62.38 ± 19.78, *p* < 0.01), had lower HR (81.23 ± 13.92 vs. 96.86 ± 22.60, *p* < 0.001), lower RR (15.00 bpm [13.00, 18.00] vs. 20.00 bpm [16.00, 24.00], *p* < 0.001), lower MAP (77.28 ± 14.18 vs. 81.88 ± 18.32, *p* < 0.001), and higher SpO_2_ (98.65 ± 3.10 vs. 95.88 ± 6.94, *p* < 0.001). Given laboratory tests, the results showed that patients in the aspirin group seemed to have shorter APTT (33.20 [29.60, 39.88] vs. 36.20 [30.87, 47.70], *p* < 0.001), lower INR (1.60 [1.50, 1.80] vs. 1.80 [1.50, 2.50], *p* < 0.001), lower BUN (17.00 [13.00, 24.00] vs. 25.00 [15.00, 44.00], *p* < 0.001), and Cr (0.90 [0.70, 1.20] vs. 1.30 [0.90, 2.00], *p* < 0.001). Compared to the non-aspirin group, the aspirin group had a higher likelihood of receiving mechanical ventilation (469 of 758 [61.9%] vs. 207 of 436 [47.5%], *p* < 0.001) and a lower likelihood of using vasopressor therapy (160 of 758 [21.1%] vs. 194 of 436 [44.5%], *p* < 0.001) and CRRT (6 of 436 [0.8%] vs. 36 of 758 [8.3%], *p* < 0.001). Regarding comorbidities, aspirin-group patients had a higher risk of hypertension (567 of 758 [74.8%] vs. 224 of 436 [51.4%], *p* < 0.001) and CAD (488 of 758 [64.4%] vs. 57 of 436 [13.1%]) and were less likely to suffer from AKI (555 of 758 [73.2%] vs. 329 of 436 [75.4%], *p* < 0.001). There were also noticeable differences in the SIC and SOFA scores between the two groups, as presented in SIC (4.82 ± 0.64 vs. 5.03 ± 0.66, *p* < 0.001) and SOFA (3.95 ± 1.95 vs. 4.62 ± 2.54, *p* < 0.001). PSM successfully matched 280 patients without aspirin and 280 with aspirin. Table 2 indicates that after PSM, parameters largely aligned with before PSM results, showing significantly lower HR (82.85 ± 15.91 vs. 94.83 ± 21.80, *p* < 0.001), RR (16.00 bpm [14.00, 19.00] vs. 19.00 bpm [16.00, 23.25], *p* < 0.001), and MAP (76.75 ± 14.79 vs. 83.07 ± 18.10, *p* < 0.001), alongside higher SpO_2_ (98.27 ± 3.65 vs. 96.15 ± 7.12, *p* < 0.001) in the aspirin group compared with the non-aspirin group. Additionally, compared with the non-aspirin group, BUN (17.00 [13.00, 26.00] vs. 23.00 [15.00, 42.00], *p* < 0.001) and Cr (0.90 [0.70, 1.40] vs. 1.25 [0.90, 1.80], *p* < 0.001) were lower in the aspirin group, with an increased prevalence of hypertension (201 of 280 [71.8%] vs. 154 of 280 [55.0%], *p* < 0.001) in the aspirin group compared with the non-aspirin group.

**TABLE 1:** Baseline characteristics of patients with SIC.

Variables	Non-aspirin group (n = 436)	Aspirin group (n = 758)	P-value
Demographic			
Age, years, mean (SD)	62.38 (19.78)	69.40 (12.18)	<0.001
Male, n (%)	232 (53.2)	511 (67.4)	<0.001
Race, n (%)			<0.001
White	266 (61.0)	541 (71.4)	
Black	54 (12.4)	44 (5.8)	
Others	116 (26.6)	173 (22.8)	
Vital signs			
Heart rate, bpm, mean (SD)	96.86 (22.60)	81.23 (13.92)	<0.001
RR, beats/min, median (IQR)^*^	20.00 [16.00, 24.00]	15.00 [13.00, 18.00]	<0.001
SpO2 (%), mean (SD)	95.88 (6.94)	98.65 (3.10)	<0.001
MAP, mmHg, mean (SD)	81.88 (18.32)	77.28 (14.18)	<0.001
Laboratory tests			
WBC count (10^3^/μL), median (IQR)^*^	9.75 [5.40, 15.10]	10.25 [7.50, 13.88]	0.068
Hgb, g/dL, mean (SD)	9.92 (2.40)	9.81 (2.13)	0.413
PLT (10^3^/μL), median (IQR)^*^	102.50 [79.00, 127.00]	115.00 [97.00, 133.00]	<0.001
BUN, mg/dL, median (IQR)^*^	25.00 [15.00, 44.00]	17.00 [13.00, 24.00]	<0.001
Cr, mg/dL, median (IQR)^*^	1.30 [0.90, 2.00]	0.90 [0.70, 1.20]	<0.001
Lactate, mmol/L, median (IQR)^*^	2.30 [1.50, 4.20]	2.20 [1.50, 3.00]	0.002
PT, seconds, median (IQR)^*^	19.25 [16.80, 26.95]	17.30 [16.40, 19.10]	<0.001
APTT, seconds, median (IQR)^*^	36.20 [30.87, 47.70]	33.20 [29.60, 39.88]	<0.001
INR, median (IQR)^*^	1.80 [1.50, 2.50]	1.60 [1.50, 1.80]	<0.001
Comorbidities			
Hypertension, n (%)	224 (51.4)	567 (74.8)	<0.001
Diabetes mellitus, n (%)	104 (23.9)	250 (33.0)	0.001
CAD, n (%)	57 (13.1)	488 (64.4)	<0.001
CKD, n (%)	83 (19.0)	142 (18.7)	0.958
AKI			<0.001
Stage I, n (%)	83 (19.0)	188 (24.8)	
Stage II, n (%)	131 (30.0)	282 (37.2)	
Stage III, n (%)	115 (26.4)	85 (11.2)	
SIC, mean (SD)	5.03 (0.66)	4.82 (0.64)	<0.001
SOFA, mean (SD)	4.62 (2.54)	3.95 (1.95)	<0.001
Treatments			
MV, n (%)	207 (47.5)	469 (61.9)	<0.001
CRRT, n (%)	36 (8.3)	6 (0.8)	<0.001
Vasopressor, n (%)	194 (44.5)	160 (21.1)	<0.001
Outcomes			
ICU stay, days, median (IQR)^*^	2.89 [1.50, 6.44]	2.17 [1.30, 3.98]	<0.001
28-day mortality, n (%)	135 (31.0)	62 (8.2)	<0.001
90-day mortality, n (%)	151 (34.6)	88 (11.6)	<0.001
1-year mortality, n (%)	181 (41.5)	125 (16.5)	<0.001

^*^Mann–Whitney U test.

Abbreviations: AKI, acute kidney injury; APTT, activated partial thromboplastin time; BUN, blood urea nitrogen; CAD, coronary artery disease; CKD, chronic kidney disease; Cr, creatinine; CRRT, continuous renal replacement therapy; Hgb, hemoglobin; ICU, intensive care unit; INR, international normalized ratio; IQR, interquartile range; MAP, mean arterial pressure; MV, mechanical ventilation; PLT, platelet count; PSM, propensity score matching; PT, prothrombin time; RR, respiratory rate; SpO_2_, saturation of peripheral oxygen; SD, standardized differences; SIC, sepsis-induced coagulopathy; SOFA, sequential organ failure assessment; WBC, white blood cell.

**TABLE 2:** Baseline characteristics of patients with SIC after PSM.

Variables	Non-aspirin group (n = 280)	Aspirin group (n = 280)	P-value	SMD
Demographic				
Age, mean (SD)	67.42 (17.75)	67.47 (13.42)	0.972	0.003
Male (%)	162 (57.9)	170 (60.7)	0.547	0.058
race (%)			0.325	0.127
White	185 (66.1)	196 (70.0)		
Black	31 (11.1)	21 (7.5)		
Others	64 (22.9)	63 (22.5)		
Vital signs				
HR, bpm, mean (SD)	94.83 (21.80)	82.85 (15.91)	<0.001	0.627
RR, beats/min, median (IQR)*	19.00 [16.00, 23.25]	16.00 [14.00, 19.00]	<0.001	0.557
SpO2 (%), mean (SD)	96.15 (7.12)]	98.27 (3.65)	<0.001	0.375
MAP, mmHg, mean (SD)	83.07 (18.10)	76.75 (14.79)	<0.001	0.382
Laboratory tests				
WBC count (10^3^/μL), median (IQR)*	9.50 [5.20, 13.50]	10.70 [7.88, 14.30]	<0.001	0.003
Hgb, g/dL, mean (SD)	10.06 (2.36)	9.94 (2.34)	0.546	0.051
PLT (10^3^/μL), median (IQR)*	106.50 [82.75, 128.00]	111.00 [92.75, 132.00]	0.009	0.271
BUN, mg/dL, median (IQR)*	23.00 [15.00, 42.00]	17.00 [13.00, 26.00]	<0.001	0.085
Cr, mg/dL, median (IQR*	1.25 [0.90, 1.80]	0.90 [0.70, 1.40]	<0.001	0.331
Lac, mmol/L, median (IQR)*	2.10 [1.40, 3.40]	2.20 [1.50, 3.30]	0.436	0.029
PT, seconds, median (IQR)*	18.60 [16.60, 25.33]	17.50 [16.58, 19.80]	0.003	0.12
APTT, seconds, median (IQR)*	35.00 [30.60, 47.18]	34.35 [30.28, 41.38]	0.111	0.193
INR, median (IQR)*	1.70 [1.50, 2.40]	1.60 [1.50, 1.80]	0.004	0.12
Comorbidities				
Hypertension, n (%)	154 (55.0)	201 (71.8)	<0.001	0.354
Diabetes mellitus, n (%)	70 (25.0)	76 (27.1)	0.63	0.049
CAD, n (%)	56 (20.0)	72 (25.7)	0.131	0.136
CKD, n (%)	56 (20.0)	53 (18.9)	0.831	0.027
AKI	72 (25.7)	83 (29.6)	0.025	0.26
Stage I, n (%)	52 (18.6)	63 (22.5)		
Stage II, n (%)	93 (33.2)	98 (35.0)		
Stage III, n (%)	63 (22.5)	36 (12.9)		
SIC, median (IQR)*	5.00 [4.00, 5.00]	5.00 [4.00, 5.00]	0.654	0.032
SOFA, median (IQR)*	4.00 [3.00, 5.00]	4.00 [2.00, 5.00]	0.428	0.082
Treatments				
MV, n (%)	138 (49.3)	148 (52.9)	0.447	0.071
CRRT, n (%)	4 (1.4)	6 (2.1)	0.75	0.054
Vasopressor, n (%)	90 (32.1)	87 (31.1)	0.856	0.023
Outcomes				
ICU stay, days, median (IQR)*	3.14 [1.65, 6.75]	2.19 [1.29, 4.14]	<0.001	0.206
28-day mortality, n (%)	82 (29.3)	33 (11.8)	<0.001	0.444
90-day mortality, n (%)	94 (33.6)	47 (16.8)	<0.001	0.394
1-year mortality, n (%)	118 (42.1)	62 (22.1)	<0.001	0.438

*Mann–Whitney U test.

Abbreviations: AKI, acute kidney injury; APTT, activated partial thromboplastin time; BUN, blood urea nitrogen; CAD, coronary artery disease; CKD, chronic kidney disease; Cr, creatinine; CRRT, continuous renal replacement therapy; Hgb, hemoglobin; ICU, intensive care unit; INR, international normalized ratio; IQR, interquartile range; MAP, mean arterial pressure; MV, mechanical ventilation; PLT, platelet count; PSM, propensity score matching; PT, prothrombin time; RR, respiratory rate; SpO_2_, saturation of peripheral oxygen; SD, standardized differences; SIC, sepsis-induced coagulopathy; SOFA, sequential organ failure assessment; WBC, white blood cell.

Before PSM, aspirin-treated patients exhibited significantly lower mortality rates at 28 days (62 of 758 [8.2%] vs. 135 of 436 [31.0%], *p* < 0.001), 90 days (88 of 758 [11.6%] vs. 151 of 436 [34.6%], *p* < 0.001), and 1 year (125 of 758 [16.5%] vs. 181 of 436 [41.5%], *p* < 0.001), as well as a reduced length of ICU stay (2.17 days [1.30, 3.98] vs. 2.89 days [1.50, 6.44], *p* < 0.001), compared to those not receiving aspirin. In the PSM cohort, the aspirin group exhibited significantly lower mortality rates at 28 days (33 of 280 [11.8%] vs. 82 of 280 [29.3%], *p* < 0.001), 90 days (47 of 280 [16.8%] vs. 94 of 280 [33.6%], *p* < 0.001), and 1 year (62 of 280 [22.1%] vs. 118 of 280 [42.1%], *p* < 0.001), along with a reduced ICU stay duration (2.19 days [1.29, 4.14] vs. 3.14 days [1.65, 6.75], *p* < 0.001).

### 3.2 Association between aspirin use and mortality

**FIGURE 2:**
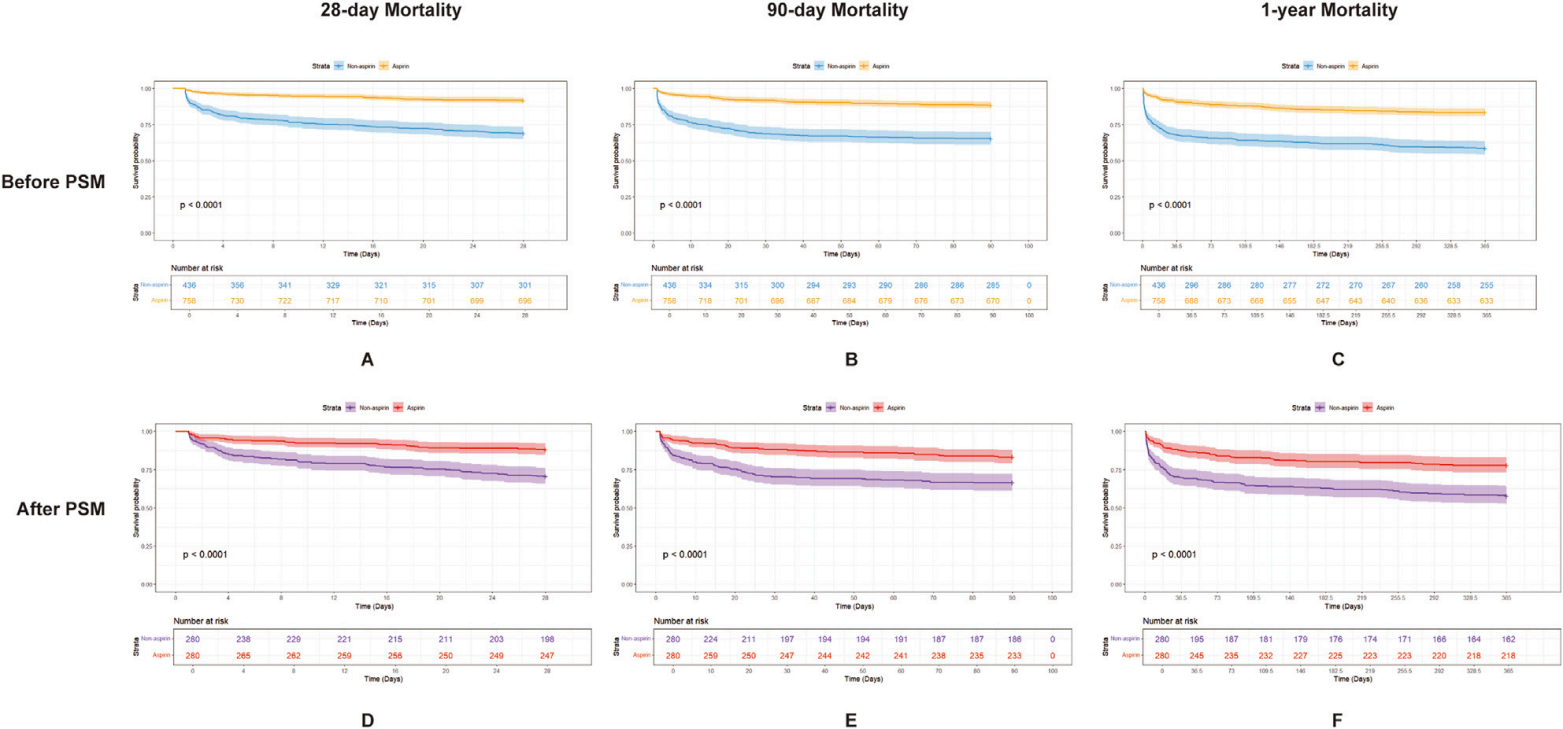
Kaplan–Meier survival curves for 28-day, 90-day, and 1-year mortality in patients with SIC, comparing the two groups (aspirin and non-aspirin) before and after PSM. **(A–C)** show the 28-day, 90-day, and 1-year mortality, respectively, in patients with SIC before PSM. Aspirin users are represented by the orange line and non-aspirin users by the blue line. **(D–F)** show the 28-day, 90-day, and 1-year mortality, respectively, in patients with SIC after PSM. Aspirin users are represented by the red line and non-aspirin users by the purple line. Abbreviations: PSM, propensity score matching; SIC, sepsis-induced coagulopathy.

Figure 2 presents Kaplan–Meier curves illustrating 28-day, 90-day, and 1-year mortality based on aspirin use in the cohort, both before and after PSM. The data revealed that the aspirin group consistently exhibited significantly higher survival rates across all time points compared to the non-aspirin group, regardless of PSM. Univariate Cox regression analyses identified risk factors linked to mortality at 28 days, 90 days, and 1 year, both before and after PSM (Supplementary Table S2). RR, heart rate, SpO_2_, Cr, lactate, AKI, and CKD were associated with increased mortality rates at 28 days, 90 days, and 1 year. Those variables from univariate analysis with p-values less than 0.05 that were applicable in clinical practice were included in the multivariate analysis. Multivariate Cox regression analyses evaluated the impact of variables on mortality at 28 days, 90 days, and 1 year (Supplementary Table S3). The study found that aspirin therapy significantly reduced mortality at 28 days, 90 days, and 1 year in both before and after PSM cohorts, with HR of 0.48 (95% CI: 0.32–0.71, *p =* 0.0003) vs. 0.45 (95% CI: 0.29–0.7, *p* = 0.0004); 0.59 (95% CI: 0.42–0.85, *p =* 0.0039) vs. 0.55 (95% CI: 0.37–0.81, *p =* 0.0025); and 0.64 (95% CI: 0.47–0.87, *p =* 0.0042) vs. 0.59 (95% CI: 0.42–0.83, *p =* 0.0028), respectively (Table 3).

**TABLE 3:** Association between aspirin therapy and mortality in patients with SIC.

	Before PSM				After PSM			
	Univariable analysis		Multivariable analysis		Univariable analysis		Multivariable analysis	
Outcomes	HR	P value	HR	P value	HR	P value	HR	P value
28-day mortality	0.23 (0.17–0.31)	<0.001	0.48 (0.32–0.71)	0.0003	0.36 (0.24–0.54)	<0.001	0.45 (0.29–0.7)	0.0004
90-day mortality	0.28 (0.22–0.37)	<0.001	0.59 (0.42–0.85)	0.0039	0.44 (0.31–0.63)	<0.001	0.55 (0.37–0.81)	0.0025
1-year mortality	0.32 (0.26–0.41)	<0.001	0.64 (0.47–0.87)	0.0042	0.45 (0.33–0.61)	<0.001	0.59 (0.42–0.83)	0.0028

Abbreviations: HR, hazard ratio; PSM, propensity score matching; SIC, sepsis-induced coagulopathy.

### 3.3 Subgroup analysis

**FIGURE 3:**
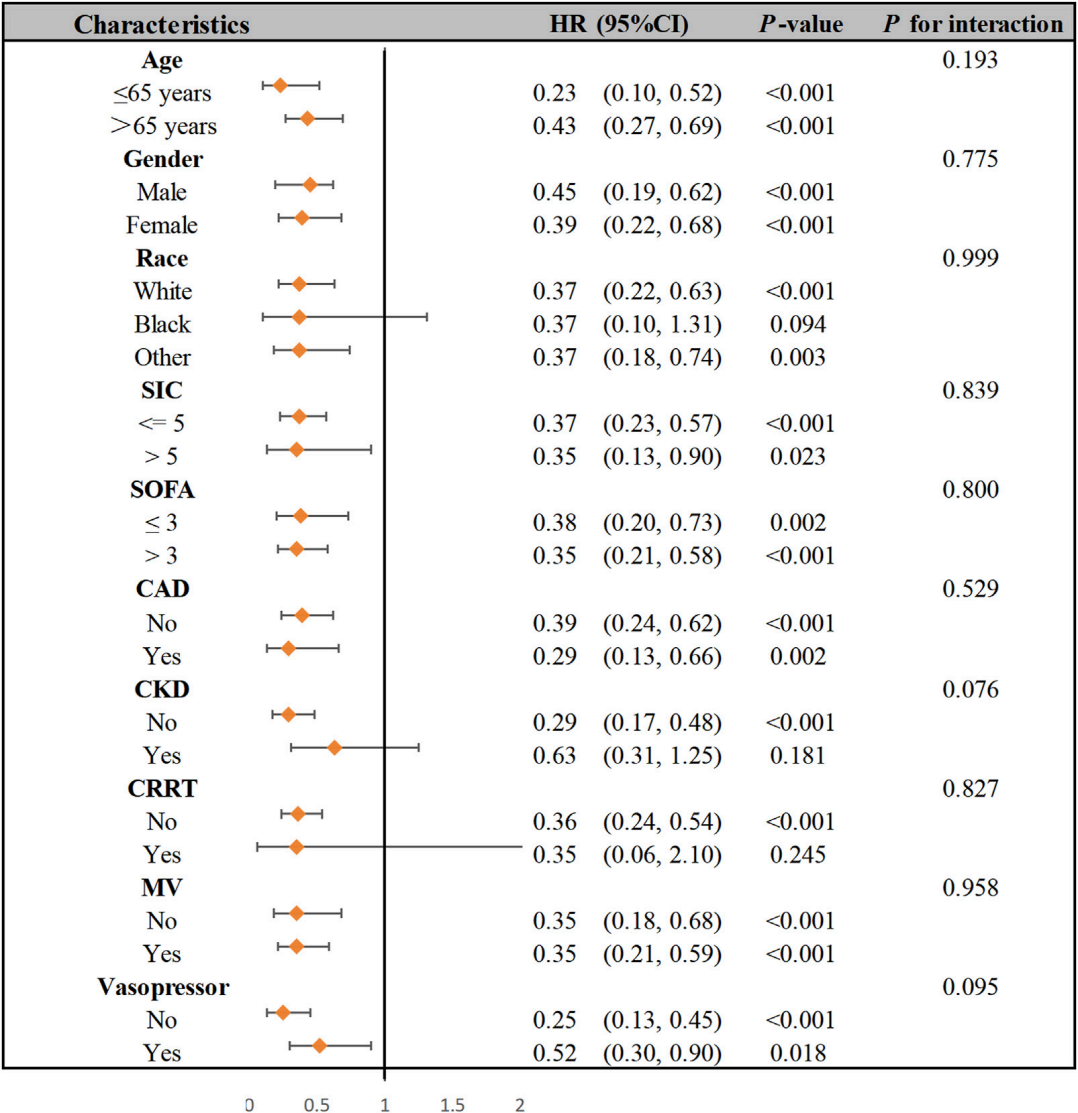
Subgroup analysis of the association between aspirin therapy and 28-day hospital mortality in patients with SIC. Abbreviations: SIC, sepsis-induced coagulopathy; SOFA, sequential organ failure assessment; CAD, coronary artery disease; CKD, chronic kidney disease; CRRT, continuous renal replacement therapy; MV, mechanical ventilation; HR, hazard ratio.

The SIC patients were stratified into subgroups based on the following: age ≥65 years (cut-off), SIC score ≥5 (cut-off), SOFA score ≥3 (cut-off), CAD, CKD, CRRT, MV, and vasopressor use. Figure 3 presents a forest plot depicting aspirin’s impact on 28-day mortality in SIC patients. No significant interaction was found between these subgroups in terms of 28-day mortality. The relationship between aspirin administration and 28-day mortality in SIC patients remained robust and reliable when subgroups were analyzed.

### 3.4 Dose-response relationship between aspirin administration and survival outcomes

**FIGURE 4:**
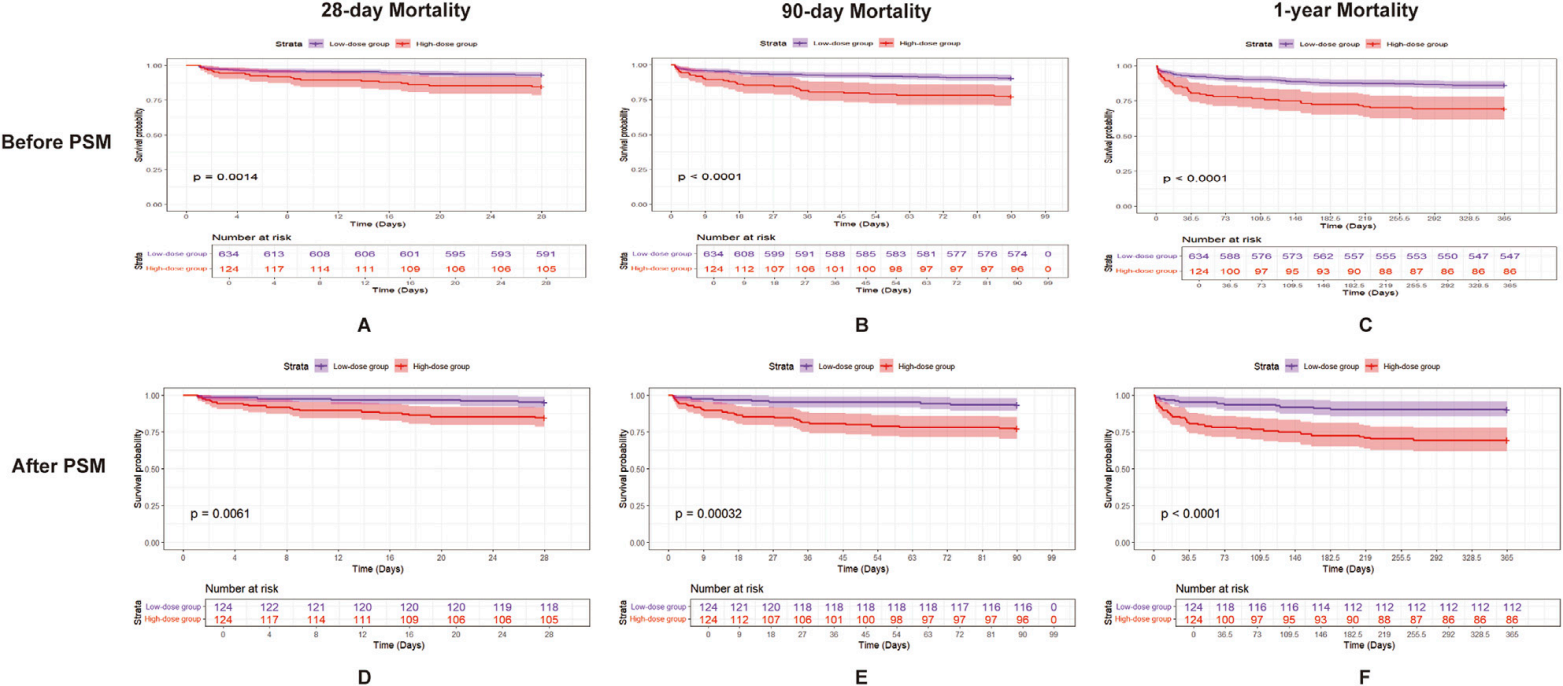
Kaplan–Meier survival curves for 28-day, 90-day, and 1-year mortality in patients with SIC, comparing the two groups (low-dose aspirin and high-dose aspirin) before and after PSM. **(A–C)** show the 28-day, 90-day, and 1-year mortality, respectively, in patients with SIC before PSM, and **(D–F)** show the 28-day, 90-day, and 1-year mortality, respectively, in patients with SIC after PSM. Low-dose aspirin users are represented by the purple line and high-dose aspirin users by the red line. Abbreviations: PSM, propensity score matching; SIC, sepsis-induced coagulopathy.

To assess the protective effects of aspirin dosage in SIC patients, we divided them into two groups: low-dose (n = 634) and high-dose (n = 124), based on whether their aspirin intake exceeded 81 mg/day (Davidson et al., 2021). Patients in the lower-dose group exhibited significantly lower Cr levels (0.90 [0.70, 1.20] vs. 1.00 [0.80, 1.40], *p* < 0.01), reduced 28-day mortality (43 of 634 [6.8%] vs. 19 of 124 [15.3%], *p* = 0.03), 90-day mortality (60 of 634 [9.5%] vs. 28 of 124 [22.6%], *p* < 0.01), 1-year mortality (87 of 634 [13.7%] vs. 38 of 124 [30.6%], *p* < 0.01), and a shorter ICU stay (2.12 days [1.29, 3.46] vs. 2.95 days [1.46, 5.88], *p* < 0.01) compared to the high-dose group (Supplementary Table S4). After PSM, patients in the lower-dose group exhibited significantly lower 28-day mortality (6 of 124 [4.8%] vs. 19 of 124 [15.3%], *p* = 0.011), 90-day mortality (8 of 124 [6.5%] vs. 28 of 124 [22.6%], *p* = 0.01), and 1-year mortality (12 of 124 [9.7%] vs. 38 of 124 [30.6%], *p* < 0.01) compared to the high-dose group. Kaplan-Meier analysis revealed that the low-dose group exhibited significantly higher survival rates at 28 days, 90 days, and 1 year compared to the high-dose group (*p* = 0.0014, *p* < 0.0001, *p* < 0.0001, respectively) before PSM. After PSM, Kaplan-Meier analysis revealed similar results (*p* = 0.0061, *p* < 0.00032, *p* < 0.0001, respectively) (Figure 4).

### 3.5 External validation

**FIGURE 5:**
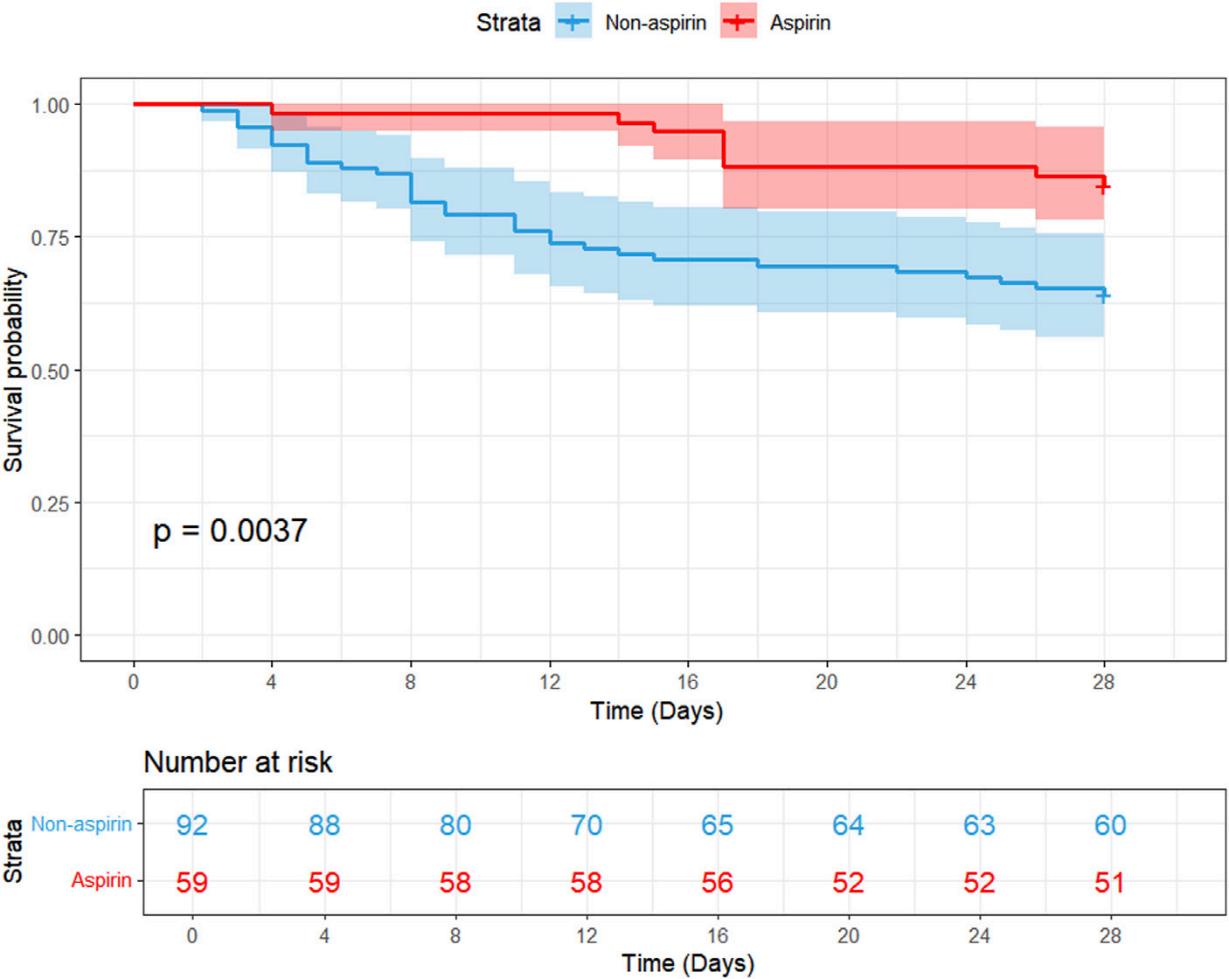
Kaplan–Meier survival curves comparing 28-day mortality between the two groups (aspirin and non-aspirin) in the external cohort of patients with SIC. Aspirin users are represented by the red line, and non-aspirin users are represented by the blue line. Abbreviations: PSM, propensity score matching; SIC, sepsis-induced coagulopathy.

A study conducted from January 2021 to May 2024 at Qilu Hospital of Shandong University involved 151 ICU patients with SIC to investigate the relationship between aspirin treatment and 28-day mortality. We classified ICU-admitted patients with SIC from Qilu Hospital of Shandong University into the aspirin group (n = 59) and the non-aspirin group (n = 92). The two groups showed significant differences in heart rate (80.00 bpm [71.00, 84.50] vs. 94.00 bpm [78.00, 109.00], *p* < 0.001), respiratory rate (15.00 bpm [13.00, 18.00] vs. 20.00 bpm [16.00, 27.00], *p* < 0.001), and 28-day mortality (9 of 59 [15.3%] vs. 33 of 92 [35.9%], *p* = 0.01). Compared with the non-aspirin group, patients in the aspirin group had higher SpO_2_ (100.00 [98.00, 100.00] vs. 98.00 [95.00, 100.00], *p* = 0.01), lower Cr level (0.90 [0.70, 1.40] vs. 1.30 [0.88, 2.30], *p* = 0.02), and lower usage rates of vasopressors (15 of 59 [25.4%] vs. 39 of 92 [42.4%], *p* = 0.051) (Supplementary Table S5). We examined the link between aspirin use and 28-day mortality employing both univariate and multivariate Cox regression analyses. Multivariable Cox regression analysis (Supplementary Table S6) indicates that aspirin therapy could serve as a protective intervention for SIC patients (HR: 0.37, 95% CI: 0.16–0.88, *p* = 0.0243). According to the Kaplan–Meier curve (Figure 5), the 28-day survival rate in the aspirin group was significantly higher than that in the non-aspirin group (log-rank test: *p* = 0.0037).

## 4 Discussion

Our retrospective study revealed a positive association between aspirin administration and the survival rates of patients with SIC. Low-dose aspirin users had a better prognosis than high-dose users. In the external validation cohort, our study showed that patients with SIC receiving aspirin exhibited a notable decrease in mortality.

Our study’s findings align with numerous other studies indicating that antiplatelet drugs can lower sepsis-related mortality. A national cohort study involving 683,421 sepsis patients found that antiplatelet drugs, primarily aspirin, significantly reduced in-hospital mortality (Tsai et al., 2015). Additionally, a study indicated that aspirin use in patients with sepsis-induced myocardial injury was associated with reduced mortality rates at 28 days, 90 days, and 1 year, highlighting its beneficial impact on patient outcomes (Dong et al., 2024). Another observational study highlighted that sepsis-related AKI may benefit from taking aspirin, demonstrating a notable improvement in survival rates and a shorter length of ICU stay in those receiving aspirin treatment (Chen S. et al., 2023). While our study found a statistically significant reduction in ICU length of stay with aspirin use (median 3.14 vs. 2.19 days, *p <* 0.001), the clinical significance of such a difference of less than 1 day is worthy of careful consideration. This moderate reduction may have a limited impact on hospital operations or patient prognosis, especially considering that the length of stay in the ICU for both groups of patients was relatively short. The finding may reflect our study’s smaller sample size (n = 280) compared to larger retrospective analyses (Chen J. et al., 2023), where more substantial differences were observed. However, even small reductions could be meaningful for individual high-risk patients or during ICU capacity constraints. Other studies have demonstrated that patients undergoing antiplatelet therapy had a significantly reduced mortality rate compared to those who do not receive such treatment (Hamid et al., 2017; Jain et al., 2022). These findings align with a narrative review that discusses the potential role of aspirin, among other therapies, in improving mortality rates in patients with sepsis on mechanical ventilation (Al-Husinat et al., 2023). The evidence suggests that aspirin may improve survival rates in sepsis patients and could be considered a therapeutic option for those with SIC, primarily owing to its ability to release proinflammatory cytokines and its properties of antiplatelet aggregation, which can alleviate coagulation disorders to some extent (Ikonomidis et al., 1999; Vane and Botting, 2003).

The activation of coagulation and platelets, as well as inflammatory cells, and damage to vascular endothelial is the main pathway during the progression of SIC induced by infection. While there are some causes leading to DIC, sepsis is one of them. In clinical practice, patients who develop DIC have already reached the terminal and irreversible stage of coagulation dysfunction. Criteria for SIC diagnosis proposed by the International Society on Thrombosis and Haemostasis are used to identify septic patients before DIC occurs. Early intervention in the disease may bring benefits to these patients (Iba et al., 2017). The interplay between coagulation and inflammation is critical because SIC can result in DIC, a severe complication that further complicates patient management (Wei et al., 2024). The mechanism of aspirin in coagulopathy is complex and involves multiple aspects, including inhibition of platelet aggregation, regulation of coagulation factors, and protection against endothelial dysfunction (Mekaj et al., 2015). Patients with SIC may benefit from aspirin administration. Studies have shown that aspirin can reduce the risk of thromboembolic events without significantly increasing the risk of major bleeding in certain patient populations (Dugani et al., 2018; Mogul et al., 2021). Moreover, the personalized prediction of cardiovascular benefits and bleeding harms from aspirin has been explored in various studies. For instance, a benefit-harm analysis conducted in New Zealand demonstrated that aspirin could result in a net benefit for individuals with higher baseline cardiovascular risk and lower bleeding risk (Selak et al., 2019). This approach can be extrapolated to SIC management, where the assessment of individual thrombotic and hemorrhagic risks can guide the use of aspirin as part of a tailored therapeutic strategy. Early administration of anticoagulants such as the use of unfractionated heparin reduces in-hospital mortality among patients with moderate SIC (Ushio et al., 2024). This suggests that an approach similar to that of using aspirin could be beneficial, particularly in patients with thrombosis and bleeding risks due to the complex nature of SIC. In conclusion, while further research is necessary to validate aspirin’s safety and efficacy, its potential benefits for SIC patients’ survival outcomes warrant further clinical investigation.

In contrast to the significant mortality reduction observed with low-dose aspirin, our study found that high-dose aspirin did not demonstrate similar benefits in SIC patients. This phenomenon provides deep insight into the role of aspirin in SIC management. Low-dose aspirin (75–81 mg/day) (Eisen, 2012) selectively inhibits platelet COX-1, reducing thromboxane A2 (TXA2) without significantly affecting endothelial COX-2-derived prostacyclin (PGI2) (Badimon et al., 2021). In contrast, high-dose aspirin non-selectively inhibits both COX-1 and COX-2, potentially impairing PGI2-mediated vasodilation and anti-thrombotic effects (Patrono et al., 2005). In SIC, where endothelial dysfunction is prominent, this imbalance may exacerbate microthrombosis (Dechamps et al., 2021). Furthermore, research on patients undergoing percutaneous coronary intervention (PCI) has shown that low-dose aspirin is associated with a lower risk of bleeding events compared to high-dose aspirin, without compromising the efficacy in preventing major adverse cardiovascular events (MACE) (Xian et al., 2015). However, our study lacked granular bleeding data; prior trials in critical illness suggest that high-dose aspirin increases the gastrointestinal bleeding risk (Chen J. et al., 2023), which may counterbalance mortality benefits in vulnerable SIC patients. Our dose analysis was exploratory, and the high-dose group sample size may limit statistical power. Additionally, unmeasured confounders (e.g., bleeding events and drug compliance) could influence results. Prospective trials are needed to validate this dose-response relationship. In conclusion, physicians should weigh the potential benefits and risks of high-dose aspirin to optimize treatment options and improve survival.

This study has some limitations. First, despite the strict PSM, the possibility of residual confounding factors still exists. Aspirin may be prescribed based on clinical indications, which are not recorded in the database, such as the doctor’s judgment or unrecorded comorbidities. These unmeasured factors may affect the likelihood of patients receiving aspirin treatment and their outcomes. Therefore, although the observed association is statistically robust, it still needs to be interpreted with caution. Second, we conducted subgroup analyses based on age, race, gender, intervention, comorbidity, SOFA, and SIC score. The p-values for interactions among these subgroups indicate no significant influence on the 28-day mortality. The observed results may not uniformly apply to all subsets of patients within our population. Factors such as other antiplatelet drugs, anticoagulant drug use, etc., could potentially modify the treatment response or outcomes observed. While we acknowledge that heterogeneity represents a limitation of our study, we now explicitly emphasize the need for future research to explore these subgroup differences using subphenotype-based methodologies (Yang et al., 2024). Third, since our study relies on retrospective cohort data, some critical temporal information was not fully recorded, which prevented us from strictly anchoring the simulation process of the target trial, thereby limiting the applicability of the Target Trial Emulation (TTE) method in our analysis (Yang et al., 2025). Fourth, the MIMIC-IV database does not contain the underlying cause of death (e.g., cardiac/infectious/hemorrhagic) and only provides binary in-hospital mortality status (survival/death). Only deaths during hospitalization were recorded, and deaths after discharge were completely missing. Taking all of the above into consideration, these limitations underscore the need for a prospective, randomized controlled trial (RCT) to validate our findings. A well-designed RCT would allow for standardized aspirin dosing, rigorous control of confounders, and direct assessment of causality. Additionally, prospective studies could explore mechanistic pathways (e.g., platelet inhibition, endothelial protection) and identify patient subgroups most likely to benefit from aspirin therapy in SIC.

In summary, our study demonstrates that taking aspirin may improve the survival rate in hospitalized patients with SIC. This conclusion, however, needs to be verified by further prospective studies.

## Data Availability

The original contributions presented in the study are included in the article/Supplementary Material, further inquiries can be directed to the corresponding author.
