# Supplementary material for: Aspirin is associated with a reduction in mortality rate for patients with sepsis-induced coagulopathy: a retrospective cohort study
Source: Front Pharmacol. 2025 Jul 28;16:1537994. doi: 10.3389/fphar.2025.1537994 (PMC12336037; doi:10.3389/fphar.2025.1537994)
Supplement: Supplementary file 1 [file Supplementaryfile1.docx]

**Supplementary Table 1: Univariable Cox regression in before and after PSM cohorts**

| Variables | Before PSM | | | | | | After PSM | | | | | |
| --- | --- | --- | --- | --- | --- | --- | --- | --- | --- | --- | --- | --- |
|  | 28-day mortality | | 90-day mortality | | 1-year mortality | | 28-day mortality | | 90-day mortality | | 1-year mortality | |
|  | HR | P value | HR | P value | HR | P value | HR | P value | HR | P value | HR | P value |
| Demographic |  |  |  |  |  |  |  |  |  |  |  |  |
| Age | 1.01 (1-1.02) | 0.058 | 1.01 (1-1.02) | 0.004 | 1.02 (1.01-1.03) | <0.01 | 1.02 (1.01-1.04) | 0.001 | 1.03 (1.01-1.04) | <0.01 | 1.03 (1.02-1.04) | <0.01 |
| Gender | 0.58 (0.44-0.77) | <0.01 | 0.65 (0.5-0.84) | 0.001 | 0.74 (0.59-0.93) | 0.009 | 0.62 (0.43-0.89) | 0.011 | 0.67 (0.48-0.94) | 0.019 | 0.78 (0.58-1.05) | 0.1 |
| Race |  |  |  |  |  |  |  |  |  |  |  |  |
| Black | 1.57 (0.99-2.5) | 0.057 | 1.39 (0.91-2.15) | 0.131 | 1.24 (0.84-1.83) | 0.276 | 1.74 (0.97-3.1) | 0.062 | 1.37 (0.79-2.38) | 0.256 | 1.21 (0.74-2) | 0.443 |
| Others | 1.59 (1.17-2.17) | 0.003 | 1.33 (1-1.78) | 0.052 | 1.09 (0.83-1.42) | 0.534 | 1.94 (1.3-2.91) | 0.001 | 1.52 (1.04-2.21) | 0.029 | 1.26 (0.89-1.78) | 0.188 |
| Vital signs |  |  |  |  |  |  |  |  |  |  |  |  |
| Heart rate | 1.02 (1.01-1.03) | <0.01 | 1.02 (1.01-1.02) | <0.01 | 1.02 (1.01-1.02) | <0.01 | 1.01 (1-1.02) | 0.017 | 1.01 (1-1.02) | 0.03 | 1.01 (1-1.02) | 0.01 |
| RR | 1.07 (1.05-1.09) | <0.01 | 1.08 (1.06-1.09) | <0.01 | 1.07 (1.06-1.09) | <0.01 | 1.05 (1.02-1.08) | <0.01 | 1.06 (1.03-1.08) | <0.01 | 1.06 (1.03-1.08) | <0.01 |
| SpO2 | 0.94 (0.93-0.95) | <0.01 | 0.94 (0.93-0.95) | <0.01 | 0.94 (0.93-0.95) | <0.01 | 0.94 (0.93-0.96) | <0.01 | 0.95 (0.93-0.96) | <0.01 | 0.94 (0.93-0.96) | <0.01 |
| MAP | 1 (1-1.01) | 0.356 | 1 (1-1.01) | 0.361 | 1.01 (1-1.01) | 0.083 | 1 (0.99-1.01) | 0.538 | 1 (0.99-1.01) | 0.377 | 1 (0.99-1.01) | 0.851 |
| BMI | 1.01 (0.99-1.04) | 0.159 | 1.01 (0.99-1.03) | 0.376 | 1.01 (0.99-1.03) | 0.247 | 0.99 (0.96-1.02) | 0.603 | 0.99 (0.97-1.02) | 0.573 | 0.99 (0.97-1.02) | 0.66 |
| Laboratory tests |  |  |  |  |  |  |  |  |  |  |  |  |
| Cr | 1.37 (1.28-1.46) | <0.01 | 1.35 (1.27-1.43) | <0.01 | 1.35 (1.28-1.42) | <0.01 | 1.33 (1.21-1.46) | <0.01 | 1.31 (1.2-1.43) | <0.01 | 1.3 (1.2-1.41) | <0.01 |
| BUN | 1 (1-1) | 0.782 | 1 (1-1) | 0.762 | 1 (1-1) | 0.731 | 1 (1-1) | 0.833 | 1 (1-1) | 0.815 | 1 (1-1) | 0.793 |
| APTT | 1.01 (1-1.01) | 0.008 | 1 (1-1.01) | 0.027 | 1.01 (1-1.01) | 0.004 | 1 (1-1.01) | 0.409 | 1 (1-1.01) | 0.5 | 1 (1-1.01) | 0.437 |
| PT | 1 (1-1) | 0.527 | 1 (1-1) | 0.082 | 1 (1-1) | 0.115 | 1 (1-1) | 0.418 | 1 (1-1) | 0.495 | 1 (1-1) | 0.613 |
| INR | 1 (1-1) | 0.528 | 1 (1-1) | 0.082 | 1 (1-1) | 0.116 | 1 (1-1) | 0.418 | 1 (1-1) | 0.495 | 1 (1-1) | 0.613 |
| WBC | 1.01 (1-1.02) | 0.048 | 1.01 (1-1.02) | 0.048 | 1.01 (1-1.02) | 0.003 | 0.99 (0.97-1.02) | 0.636 | 1 (0.99-1.02) | 0.87 | 1.01 (1-1.02) | 0.061 |
| PLT | 1 (0.99-1) | 0.063 | 1 (0.99-1) | 0.021 | 0.99 (0.99-1) | 0.006 | 1 (0.99-1) | 0.221 | 1 (0.99-1) | 0.112 | 0.99 (0.99-1) | 0.017 |
| Hgb | 1.03 (0.97-1.1) | 0.304 | 1.01 (0.95-1.07) | 0.79 | 0.98 (0.94-1.04) | 0.551 | 1.05 (0.97-1.13) | 0.2 | 1.02 (0.96-1.1) | 0.498 | 0.99 (0.93-1.05) | 0.751 |
| Lactate | 1.26 (1.22-1.31) | <0.01 | 1.24 (1.2-1.28) | <0.01 | 1.2 (1.16-1.24) | 0<0.01 | 1.22 (1.15-1.3) | <0.01 | 1.2 (1.13-1.27) | <0.01 | 1.15 (1.08-1.21) | <0.01 |
| Comorbidities |  |  |  |  |  |  |  |  |  |  |  |  |
| CKD | 1.67 (1.22-2.29) | 0.001 | 1.88 (1.42-2.48) | <0.01 | 2.02 (1.58-2.58) | <0.01 | 1.88 (1.26-2.8) | 0.002 | 1.88 (1.31-2.7) | 0.001 | 1.87 (1.35-2.59) | <0.01 |
| CAD | 0.43 (0.31-0.59) | <0.01 | 0.51 (0.39-0.67) | <0.01 | 0.58 (0.46-0.73) | <0.01 | 1.05 (0.68-1.61) | 0.835 | 1.26 (0.87-1.83) | 0.22 | 1.27 (0.91-1.76) | 0.162 |
| DM | 1.01 (0.75-1.37) | 0.945 | 1.04 (0.79-1.37) | 0.765 | 1.2 (0.94-1.52) | 0.14 | 1.4 (0.95-2.07) | 0.092 | 1.31 (0.91-1.87) | 0.143 | 1.44 (1.05-1.97) | 0.023 |
| Hypertension | 0.91 (0.68-1.21) | 0.507 | 1.03 (0.79-1.35) | 0.812 | 1.07 (0.84-1.36) | 0.582 | 1.37 (0.92-2.04) | 0.119 | 1.53 (1.06-2.2) | 0.023 | 1.43 (1.04-1.97) | 0.027 |
| AKI |  |  |  |  |  |  |  |  |  |  |  |  |
| Stage I, n(%) | 1.84 (1.07-3.14) | 0.026 | 1.86 (1.13-3.08) | 0.015 | 1.4 (0.93-2.11) | 0.111 | 2.05 (1.1-3.82) | 0.023 | 2.01 (1.11-3.63) | 0.021 | 1.74 (1.05-2.89) | 0.03 |
| Stage II, n(%) | 1.8 (1.09-2.96) | 0.021 | 2.16 (1.37-3.4) | 0.001 | 1.76 (1.22-2.53) | 0.002 | 1.56 (0.86-2.8) | 0.14 | 1.9 (1.11-3.26) | 0.02 | 1.82 (1.16-2.85) | 0.009 |
| Stage III, n(%) | 8.27 (5.18-13.19) | <0.01 | 9.06 (5.85-14.02) | <0.01 | 6.76 (4.76-9.62) | <0.01 | 5.07 (2.88-8.9) | <0.01 | 6 (3.55-10.15) | <0.01 | 4.99 (3.18-7.82) | <0.01 |
| SIC | 1.48 (1.19-1.83) | <0.01 | 1.5 (1.23-1.82) | <0.01 | 1.35 (1.14-1.6) | 0.001 | 1.18 (0.9-1.55) | 0.226 | 1.3 (1.02-1.66) | 0.035 | 1.19 (0.96-1.47) | 0.122 |
| SOFA | 1.57 (0.99-2.5) | 0.057 | 1.2 (1.15-1.26) | <0.01 | 1.17 (1.12-1.22) | <0.01 | 1.17 (1.08-1.26) | <0.01 | 1.17 (1.08-1.25) | <0.01 | 1.12 (1.04-1.19) | 0.002 |
| Treatments |  |  |  |  |  |  |  |  |  |  |  |  |
| MV | 1.43 (1.07-1.92) | 0.016 | 1.29 (0.99-1.67) | 0.055 | 1.14 (0.9-1.42) | 0.275 | 1.54 (1.06-2.24) | 0.023 | 1.44 (1.03-2.01) | 0.033 | 1.46 (1.09-1.96) | 0.012 |
| CRRT | 6.23 (4.09-9.5) | <0.01 | 6.06 (4.08-9) | <0.01 | 5.33 (3.65-7.78) | <0.01 | 3.44 (1.4-8.44) | 0.007 | 4.25 (1.99-9.09) | <0.01 | 4.15 (2.04-8.44) | <0.01 |
| Vasopressor | 4.11 (3.1-5.47) | <0.01 | 3.56 (2.76-4.6) | <0.01 | 3.02 (2.41-3.78) | <0.01 | 2.32 (1.61-3.34) | <0.01 | 2 (1.44-2.8) | <0.01 | 1.89 (1.4-2.54) | <0.01 |

**Abbreviations:** PSM, propensity score matching; RR, respiratory rate; SpO2, saturation of peripheral oxygen; MAP, mean arterial pressure; BMI, body mass index; Cr, creatinine; BUN, blood urea nitrogen; APTT, activated partial thromboplastin time; PT, prothrombin time; INR, international normalized ratio; WBC, white blood cell; PLT, platelet count; Hgb, hemoglobin; CKD, chronic kidney disease; CAD, coronary artery disease; DM, diabetes mellitus; AKI, acute kidney injury; MV, mechanical ventilation; CRRT, continuous renal replacement therapy; SIC, sepsis-induced coagulopathy; SOFA, sequential organ failure assessment; HR, hazard ratio

**Supplementary Table 2:** **Multivariable Cox regression in before and after PSM cohorts**

| Variables | Before PSM | | | | | | After PSM | | | | | |
| --- | --- | --- | --- | --- | --- | --- | --- | --- | --- | --- | --- | --- |
|  | 28-day mortality | | 90-day mortality | | 1-year mortality | | 28-day mortality | | 90-day mortality | | 1-year mortality | |
|  | HR | P value | HR | P value | HR | P value | HR | P value | HR | P value | HR | P value |
| Demographic |  |  |  |  |  |  |  |  |  |  |  |  |
| Age | 1.02(1.01 - 1.03) | 0.0001 | 1.02(1.01 - 1.03) | <0.01 | 1.03(1.02 - 1.03) | <0.01 | 1.02(1.01 - 1.03) | 0.0051 | 1.02(1.01 - 1.04) | 0.0005 | 1.02(1.01 - 1.04) | <0.01 |
| Gender | 0.67(0.5 - 0.9) | 0.0075 | 0.7(0.54 - 0.92) | 0.0097 | 0.78(0.62 - 0.99) | 0.0447 | 0.6(0.41 - 0.88) | 0.0093 | 0.64(0.45 - 0.9) | 0.0106 | 0.73(0.54 - 0.99) | 0.0458 |
| Race |  |  |  |  |  |  |  |  |  |  |  |  |
| Black | 0.61(0.36 - 1.04) | 0.0687 | 0.56(0.34 - 0.93) | 0.0237 | 0.545(0.35 - 0.85) | 0.0082 | 1.1(0.57 - 2.14) | 0.7698 | 0.86(0.46 - 1.61) | 0.6423 | 0.7(0.4 - 1.23) | 0.2094 |
| Others | 1.46(1.06 - 2.02) | 0.0216 | 1.3(0.96 - 1.76) | 0.0879 | 1.09(0.82 - 1.43) | 0.5542 | 2.09(1.37 - 3.18) | 0.0006 | 1.72(1.16 - 2.54) | 0.0069 | 1.39(0.97 - 1.98) | 0.0728 |
| Vital signs |  |  |  |  |  |  |  |  |  |  |  |  |
| Heart rate | 1(1 - 1.01) | 0.3087 | 1(1 - 1.01) | 0.2062 | 1(1 - 1.01) | 0.2033 | 1(0.99 - 1.01) | 0.8986 | 1(0.99 - 1.01) | 0.7587 | 1(0.99 - 1.01) | 0.8454 |
| RR | 1.03(1 - 1.05) | 0.0274 | 1(1 - 1.01) | 0.8045 | 1(0.99 - 1) | 0.5532 | 1.03(0.99 - 1.06) | 0.1147 | 1.04(1.01 - 1.07) | 0.0165 | 1.03(1 - 1.06) | 0.0224 |
| SpO2 | 0.98(0.96 - 1) | 0.1111 | 0.98(0.96 - 1) | 0.1181 | 0.98(0.96 - 1) | 0.0717 | 0.99(0.95 - 1.02) | 0.4505 | 0.99(0.95 - 1.02) | 0.4165 | 0.98(0.95 - 1.01) | 0.2551 |
| Laboratory tests |  |  |  |  |  |  |  |  |  |  |  |  |
| Cr | 1.11(1 - 1.23) | 0.0411 | 1.07(0.97 - 1.19) | 0.1622 | 1.11(1.01 - 1.21) | 0.0232 | 1.08(0.9 - 1.3) | 0.3861 | 1.03(0.87 - 1.22) | 0.7109 | 1.1(0.94 - 1.27) | 0.2239 |
| Lactate | 1.14(1.08 - 1.19) | <0.01 | 1.12(1.07 - 1.17) | <0.01 | 1.09(1.04 - 1.13) | 0.0002 | 1.18(1.1 - 1.27) | <0.01 | 1.15(1.07 - 1.23) | 0.0001 | 1.09(1.02 - 1.16) | 0.0151 |
| Comorbidities |  |  |  |  |  |  |  |  |  |  |  |  |
| CKD | 1.13(0.79 - 1.61) | 0.5031 | 1.24(0.9 - 1.7) | 0.1899 | 1.31(1 - 1.73) | 0.0522 | 1.28(0.79 - 2.09) | 0.317 | 1.12(0.72 - 1.75) | 0.6098 | 1.08(0.72 - 1.6) | 0.7171 |
| CAD | 0.92(0.62 - 1.36) | 0.675 | 0.9(0.64 - 1.27) | 0.5419 | 0.91(0.67 - 1.22) | 0.51 |  |  |  |  |  |  |
| AKI |  |  |  |  |  |  |  |  |  |  |  |  |
| Stage I, n(%) | 1.54(0.89 - 2.67) | 0.1217 | 1.63(0.98 - 2.71) | 0.0622 | 1.21(0.8 - 1.85) | 0.3718 | 1.85(0.97 - 3.53) | 0.0608 | 1.89(1.03 - 3.48) | 0.0411 | 1.52(0.9 - 2.57) | 0.1154 |
| Stage II, n(%) | 1.38(0.82 - 2.3) | 0.2219 | 1.72(1.08 - 2.74) | 0.0235 | 1.41(0.97 - 2.05) | 0.072 | 1.3(0.71 - 2.39) | 0.4013 | 1.6(0.91 - 2.79) | 0.1021 | 1.51(0.94 - 2.41) | 0.0858 |
| Stage III, n(%) | 3.01(1.75 - 5.17) | 0.0001 | 3.81(2.31 - 6.28) | <0.01 | 2.96(1.96 - 4.47) | <0.01 | 1.92(0.96 - 3.82) | 0.0646 | 2.73(1.46 - 5.12) | 0.0018 | 2.38(1.37 - 4.14) | 0.002 |
| SIC | 1.48 (1.19-1.83) | <0.01 | 1.5 (1.23-1.82) | <0.01 | 1.35 (1.14-1.6) | 0.001 | 0.8(0.57 - 1.14) | 0.2192 | 0.89(0.65 - 1.22) | 0.4732 | 0.81(0.61 - 1.07) | 0.1415 |
| SOFA | 1.57 (0.99-2.5) | 0.057 | 1.2 (1.15-1.26) | <0.01 | 1.17 (1.12-1.22) | <0.01 | 1.05(0.95 - 1.16) | 0.3552 | 1.07(0.97 - 1.17) | 0.1827 | 1.03(0.95 - 1.13) | 0.4741 |
| Treatments |  |  |  |  |  |  |  |  |  |  |  |  |
| MV | 0.97(0.69 - 1.37) | 0.8702 | 0.91(0.67 - 1.23) | 0.5377 | 0.93(0.71 - 1.21) | 0.5779 | 1.010.64 - 1.58 | 0.9711 | 0.99(0.66 - 1.47) | 0.9524 | 1.17(0.82 - 1.66) | 0.3843 |
| CRRT | 1.07(0.62 - 1.86) | 0.8014 | 1.34(0.8 - 2.24) | 0.2621 | 1.38(0.86 - 2.22) | 0.187 | 2.96(1.12 - 7.81 | 0.0283 | 3.18(1.37 - 7.35) | 0.0069 | 2.74(1.24 - 6.06) | 0.0126 |
| Vasopressor | 1.69(1.2 - 2.4) | 0.003 | 1.56(1.14 - 2.13) | 0.0051 | 1.53(1.17 - 2.01) | 0.0021 | 1.36(0.87 - 2.11) | 0.1761 | 1.14(0.76 - 1.69) | 0.531 | 1.19(0.84 - 1.69) | 0.3225 |

**Abbreviations:** PSM, propensity score matching; RR, respiratory rate; SpO2, saturation of peripheral oxygen; Cr, creatinine; BUN, blood urea nitrogen; CKD, chronic kidney disease; AKI, acute kidney injury; MV, mechanical ventilation; CRRT, continuous renal replacement therapy; SIC, sepsis-induced coagulopathy; SOFA, sequential organ failure assessment; HR, hazard ratio

**Supplementary Table 3:** **Baseline characteristics of the aspirin groups (by dose) before and after PSM**

|  | Before PSM | | | | After PSM | | | |
| --- | --- | --- | --- | --- | --- | --- | --- | --- |
| Variables | Low does-aspirin group(n=634) | High dose-aspirin group(n=124) | P-value | SMD | Low does-aspirin group(n=124) | High dose-aspirin group(n=124) | P-value | SMD |
| Demographic |  |  |  |  |  |  |  |  |
| Age, years, mean (SD) | 68.91 (12.20) | 71.87 (11.84) | 0.013 | 0.246 | 71.72 (11.28) | 71.87 (11.84) | 0.917 | 0.013 |
| Male, n(%) | 426 (67.2) | 85 (68.5) | 0.849 | 0.029 | 87 ( 70.2) | 85 (68.5) | 0.671 | 0.114 |
| Race, n(%) |  |  | 0.231 | 0.175 |  |  |  |  |
| White | 446 (70.3) | 95 (76.6) |  |  | 99 ( 79.8) | 95 (76.6) | 0.671 | 0.114 |
| Black | 36 ( 5.7) | 8 ( 6.5) |  |  | 5 ( 4.0) | 8 ( 6.5) |  |  |
| Others | 152 (24.0) | 21 (16.9) |  |  | 20 ( 16.1) | 21 (16.9) |  |  |
| Vital signs |  |  |  |  |  |  |  |  |
| HR, bpm, mean (SD) | 80.84 (13.38) | 83.21 (16.34) | 0.083 | 0.159 | 78.98 (12.35) | 83.21 (16.34) | 0.022 | 0.292 |
| RR, beats/min, median (IQR)* | 80.00 [74.00, 87.00] | 81.00 [72.50, 91.00] | 0.181 | 0.335 | 15.00 [13.00, 17.00] | 16.00 [14.00, 20.00] | 0.012 | 0.296 |
| SpO2 (%), mean (SD) | 98.78 (3.07) | 97.98 (3.14) | 0.008 | 0.258 | 98.75 (3.49) | 97.98 (3.14) | 0.07 | 0.231 |
| MAP, mmHg, mean (SD) | 76.84 (13.64) | 79.49 (16.54) | 0.057 | 0.175 | 76.65 (14.55) | 79.49 (16.54) | 0.152 |  |
| Laboratory tests |  |  |  |  |  |  |  |  |
| WBC count (103/μL), median (IQR)* | 10.50 [7.60, 14.07] | 9.60 [7.25, 13.33] | 0.09 | 0.198 | 10.55 [7.97, 13.70] | 9.60 [7.25, 13.33] | 0.151 | 0.184 |
| Hgb, g/dL, mean(SD) | 9.75 (2.14) | 10.12 (2.07) | 0.077 | 0.176 | 9.99 (2.15) | 10.12 (2.07) | 0.635 | 0.06 |
| PLT (103/μL), median (IQR)* | 114.00 [96.00, 132.00] | 121.50 [99.75, 136.00] | 0.103 | 0.133 | 116.00 [97.00, 133.00] | 121.50 [99.75, 136.00] | 0.398 | 0.069 |
| BUN, mg/dL, median (IQR)* | 17.00 [13.00, 23.00] | 19.00 [14.00, 30.25] | 0.008 | 0.233 | 17.00 [13.00, 24.00] | 19.00 [14.00, 30.25] | 0.043 | 0.202 |
| Cr, mg/dL, median (IQR)* | 0.90 [0.70, 1.20] | 1.00 [0.80, 1.40] | <0.001 | 0.165 | 0.80 [0.70, 1.02] | 1.00 [0.80, 1.40] | <0.001 | 0.452 |
| Lactate, mmol/L, median (IQR)* | 2.20 [1.60, 2.90] | 2.15 [1.50, 3.20] | 0.798 | 0.102 | 2.30 [1.60, 3.10] | 2.15 [1.50, 3.20] | 0.68 | 0.028 |
| PT, seconds, median (IQR)* | 17.30 [16.40, 19.00] | 17.65 [16.30, 19.92] | 0.358 | 0.056 | 17.15 [16.30, 18.72] | 17.65 [16.30, 19.92] | 0.177 | 0.176 |
| APTT, seconds, median (IQR)* | 33.15 [29.42, 39.40] | 34.35 [30.20, 43.00] | 0.044 | 0.245 | 33.90 [30.05, 39.75] | 34.35 [30.20, 43.00] | 0.343 | 0.224 |
| INR, median (IQR)* | 1.60 [1.50, 1.80] | 1.60 [1.50, 1.80] | 0.391 | 0.056 | 1.60 [1.50, 1.70] | 1.60 [1.50, 1.80] | 0.196 | 0.204 |
| Comorbidities |  |  |  |  |  |  |  |  |
| Hypertension, n(%) | 472 (74.4) | 95 (76.6) | 0.693 | 0.05 | 88 ( 71.0) | 95 (76.6) | 0.386 | 0.129 |
| Diabetes mellitus, n(%) | 208 (32.8) | 42 (33.9) | 0.9 | 0.023 | 41 ( 33.1) | 42 (33.9) | 1 | 0.017 |
| CAD, n(%) | 406 (64.0) | 82 (66.1) | 0.732 | 0.044 | 78 ( 62.9) | 82 (66.1) | 0.691 | 0.067 |
| CKD, n(%) | 120 (18.9) | 22 (17.7) | 0.854 | 0.031 | 20 ( 16.1) | 22 (17.7) | 0.866 | 0.043 |
| AKI |  |  | 0.004 | 0.33 |  |  | 0.033 | 0.383 |
| Stage I, n(%) | 160 (25.2) | 28 (22.6) |  |  | 33 ( 26.6) | 28 (22.6) |  |  |
| Stage II, n(%) | 236 (37.2) | 46 (37.1) |  |  | 52 ( 41.9) | 46 (37.1) |  |  |
| Stage III, n(%) | 60 ( 9.5) | 25 (20.2) |  |  | 9 ( 7.3) | 25 (20.2) |  |  |
| SIC, mean (SD) | 4.82 (0.65) | 4.80 (0.58) | 0.671 | 0.043 |  |  |  |  |
| SOFA, mean (SD) | 4.00 (1.99) | 3.72 (1.70) | 0.147 | 0.15 |  |  |  |  |
| Treatments |  |  |  |  |  |  |  |  |
| MV, n(%) | 396 (62.5) | 73 (58.9) | 0.515 | 0.074 | 64 ( 51.6) | 73 (58.9) | 0.307 | 0.146 |
| CRRT, n(%) | 5 ( 0.8) | 1 ( 0.8) | 1 | 0.002 | 0 ( 0.0) | 1 ( 0.8) | 1 | 0.128 |
| Vasopressor, n(%) | 126 (19.9) | 34 (27.4) | 0.078 | 0.178 | 23 ( 18.5) | 34 (27.4) | 0.131 | 0.212 |
| Outcomes |  |  |  |  |  |  |  |  |
| ICU stay, days, median (IQR)* | 2.12 [1.29, 3.46] | 2.95 [1.46, 5.88] | <0.001 | 0.276 | 4.19 (6.50) | 5.11 (6.15) | 0.253 | 0.146 |
| 28-day mortality, n(%) | 43 ( 6.8) | 19 (15.3) | 0.003 | 0.275 | 6 ( 4.8) | 19 (15.3) | 0.011 | 0.354 |
| 90-day mortality, n(%) | 60 ( 9.5) | 28 (22.6) | <0.001 | 0.363 | 8 ( 6.5) | 28 (22.6) | 0.001 | 0.47 |
| 1-year mortality, n(%) | 87 (13.7) | 38 (30.6) | <0.001 | 0.416 | 12 ( 9.7) | 38 (30.6) | <0.001 | 0.541 |

* Mann-Whitney U test

**Abbreviations:** RR, respiratory rate; SpO2, saturation of peripheral oxygen; MAP, mean arterial pressure; WBC, white blood cell; Hgb, hemoglobin; PLT, platelet count; BUN, blood urea nitrogen; Cr, creatinine; PT, prothrombin time; APTT, activated partial thromboplastin time; INR, international normalized ratio; CAD, coronary artery disease; CKD, chronic kidney disease; AKI, acute kidney injury; MV, mechanical ventilation; CRRT, continuous renal replacement therapy; SIC, sepsis-induced coagulopathy; SOFA, sequential organ failure assessment

**Supplementary Table 4:** **Baseline characteristics of the aspirin-treated group in the validation cohort**

| Variables | Non-aspirin group(n=92) | Aspirin group(n=59) | P-value |
| --- | --- | --- | --- |
| Demographic |  |  |  |
| Age, years, mean(SD) | 63.89 (15.09) | 65.46 (13.92) | 0.807 |
| Male, n(%) | 61 (66.3) | 29 (49.2) | 0.054 |
| Vital signs |  |  |  |
| Heart rate, bpm, median (IQR)* | 94.00 [78.00, 109.00] | 80.00 [71.00, 84.50] | <0.001 |
| RR, beats/min, median (IQR)* | 20.00 [16.00, 27.00] | 15.00 [13.00, 18.00] | <0.001 |
| SpO2 (%), median (IQR)* | 98.00 [95.00, 100.00] | 100.00 [98.00, 100.00] | 0.001 |
| Laboratory tests |  |  |  |
| Cr, mg/dL, median (IQR)* | 1.30 [0.88, 2.30] | 0.90 [0.70, 1.40] | 0.002 |
| Lac, mmol/L, median (IQR)* | 2.45 [1.58, 3.95] | 2.00 [1.55, 3.00] | 0.102 |
| Comorbidities |  |  |  |
| CKD, n(%) | 19 (20.7) | 13 (22.0) | 1 |
| AKI |  |  | 0.403 |
| Stage I, n(%) | 18 (19.6) | 12 (20.3) |  |
| Stage II, n(%) | 24 (26.1) | 22 (37.3) |  |
| Stage III, n(%) | 28 (30.4) | 12 (20.3) |  |
| SIC, median (IQR)* | 5.00 [4.00, 6.00] | 4.00 [4.00, 5.00] | 0.099 |
| SOFA, median (IQR)* | 10.00 [7.00, 13.25] | 9.00 [5.00, 12.50] | 0.105 |
| Treatments |  |  |  |
| MV, n(%) | 48 (52.2) | 39 (66.1) | 0.128 |
| CRRT, n(%) | 8 ( 8.7) | 2 ( 3.4) | 0.345 |
| Vasopressor, n(%) | 39 (42.4) | 15 (25.4) | 0.051 |
| ICU stay, days, median (IQR)* | 28.00 [12.00, 28.00] | 28.00 [28.00, 28.00] | 0.002 |
| 28-day mortality | 33 (35.9) | 9 (15.3) | 0.01 |

* Mann-Whitney U test

**Abbreviations:** RR, respiratory rate; SpO2, saturation of peripheral oxygen; Cr, creatinine; CKD, chronic kidney disease; AKI, acute kidney injury; MV, mechanical ventilation; CRRT, continuous renal replacement therapy; SIC, sepsis-induced coagulopathy; SOFA, sequential organ failure assessment

**Supplementary Table 5:** Multivariable Cox regression in validation cohort

|  | HR | P |
| --- | --- | --- |
| Vital signs |  |  |
| Heart rate | 1.01(1 - 1.03) | 0.1174 |
| RR | 1(0.95 - 1.05) | 0.9593 |
| SpO2 | 1.13(1.01 - 1.26) | 0.035 |
| Laboratory tests |  |  |
| Cr | 0.94(0.68 - 1.3) | 0.7117 |
| Lactate | 1.02(0.89 - 1.17) | 0.7896 |
| Comorbidities |  |  |
| CKD | 0.74(0.31 - 1.79) | 0.5084 |
| AKI | 1.14(0.78 - 1.65) | 0.5031 |
| SIC | 1.26(0.85 - 1.87) | 0.2549 |
| SOFA | 0.96(0.88 - 1.04) | 0.2816 |
| Treatments |  |  |
| MV | 0.84(0.37 - 1.89) | 0.6734 |
| CRRT | 1.06(0.3 - 3.76) | 0.928 |
| Vasopressor | 1.84(0.88 - 3.84) | 0.1042 |
| Aspirin | 0.37(0.16 - 0.88) | 0.0243 |

**Abbreviations:** RR, respiratory rate; SpO2, saturation of peripheral oxygen; Cr, creatinine; CKD, chronic kidney disease; AKI, acute kidney injury; MV, mechanical ventilation; CRRT, continuous renal replacement therapy; SIC, sepsis-induced coagulopathy; SOFA, sequential organ failure assessment.

**Supplementary Figure 1.** Missing data patterns in the study variables.

**
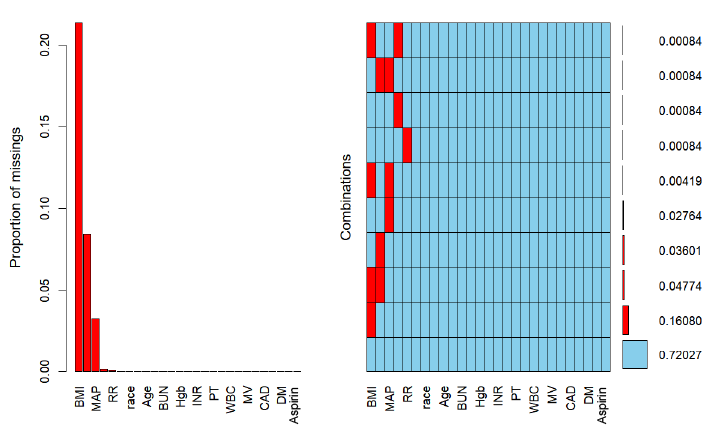
**

**Abbreviations:** BMI, Body Mass Index，RR, respiratory rate; MAP, mean arterial pressure; WBC, white blood cell; Hgb, hemoglobin; BUN, blood urea nitrogen; PT, prothrombin time; INR, international normalized ratio; CAD, coronary artery disease; DM, Diabetes Mellitus; MV, mechanical ventilation.
